# Identifying Lower Extremity Deformities in the Coronal Plane: Lessons From Remote Learning During the COVID-19 Pandemic

**DOI:** 10.7759/cureus.67684

**Published:** 2024-08-24

**Authors:** Rahul Vaidya, Kadence Rosinski, Tatiana Bunge, Daniel Cavazos, Michael S Sirkin, Anagha Purushotham, Brett Crist, Robert Teitge

**Affiliations:** 1 Department of Orthopedic Surgery, Wayne State University Detroit Medical Center, Detroit, USA; 2 Department of Orthopedic Surgery, Wayne State University School of Medicine, Detroit, USA; 3 Department of Orthopedics and Trauma, Rutgers University New Jersey Medical School, Newark, USA; 4 Department of Orthopedic Surgery, Wayne State University, Detroit, USA; 5 Department of Orthopedics and Trauma, University of Missouri, Columbia, USA

**Keywords:** joint deformity, tibial deformity, femoral deformity, total deformity, coronal alignment

## Abstract

Introduction

During the COVID-19 pandemic, the AO Foundation, an orthopedic education organization, had to transition its live education programs to an online format. Skills that were previously evaluated and corrected in person by educators were now assessed through online lectures and discussion groups. Our goal was to evaluate an online course designed to teach the skill of “leg length and coronal deformity measurement” and to assess participants’ ability to demonstrate this skill using online software.

Methods

An IRB-approved study was conducted during an online osteotomy course. A total of 176 participants were instructed on how to measure length and angulation through the course lectures and planning tutorials. The Bonesetter App (Detroit Bonesetter, Detroit, Michigan, USA) was used as the digital planning tool. Participants analyzed four cases of coronal deformity using long-leg standing radiographs. They were required to identify any deformities present, specify the type of deformity, and determine whether it affected the femur, tibia, or joint. Additionally, participants needed to calculate the deformity as part of the osteotomy planning process. Their measurements and drawings were saved and subsequently evaluated by two independent observers.

Results

Out of 176 online participants, 76% (315 out of 417) correctly completed the four exercises. The most frequent errors occurred in measuring total deformity and joint deformity. The average standard deviation of length measurements was ±0.29 cm (based on 244 measurements), while for angular measurements, it was ±0.71 degrees (based on 688 measurements). The intraclass correlation coefficient was greater than 0.96.

Conclusions

The online course effectively taught coronal deformity measurement, although participants struggled most with measuring joint angles and total deformity. Future courses should provide more detailed explanations for these measurements.

## Introduction

The skeletal geometry and limb alignment of the lower extremities play a crucial mechanical role in supporting standing, walking, and running. Limbs are designed to transfer body mass to the ground while keeping tissues within the limits of biological tolerance and minimizing biological stress [1-5]. Giovanni Alfonso Borelli (1608-1679) analyzed the function of the lower extremities by calculating the forces across muscles and joints during activities. Borelli recognized that muscles and ligaments act under great stresses several times the magnitude of body weight [1,5]. Pauwels also applied mechanical principles to understand anatomy and used the physics of columns to explain normal human coronal alignment [1,5]. The numbers generated for the normal mechanical and anatomic axes of the human lower extremity have been verified by several authors through measurements and their observations [1-5].

The mechanical axis of the human lower limb goes from the center of the hip to the center of the ankle. In a normal limb, this will pass through the knee joint just slightly medial to its center. In patients with deformity, if the axis lies lateral to the midline, the limb has a valgus alignment; if it passes medial to the midline, there is a varus alignment. The mechanical axis is three degrees medial to the vertical, which can be explained by the necessity to stand on one leg in a normal human gait. With this orientation, the alignment brings the limb under the pelvis and the center of body mass during midstance [5].

The best mechanical position for the knee joint is horizontal with the floor. With the 3-degree mechanical axis and the horizontal joint line of the knee, the distal femur has a lateral distal femur angle (LDFA) of 87 degrees to the joint line (valgus), and the tibia has an 87-degree medial proximal angle from the joint line (varus) [1-5]. On average, the anatomic axis of the femur and the mechanical axis of the femur are 6 degrees off. The mechanical axis and the anatomic axis of the tibia are the same. When there is a deformity, surgeons must determine the type of deformity (varus or valgus), the degree of deformity, and if the deformity is in the femur, tibia, joint line, or both [4,5].

While measuring coronal deformity is a skill that can be taught and learned, using digital software in a medical setting is a skill that is unfamiliar to many orthopedic surgeons. Due to the COVID-19 pandemic, many educational courses were transitioned to an online format, necessitating the development of technology to enhance the learning, testing, and verification that participants understood and captured the skills that were taught. As a result, AO North America developed an online osteotomy course, and The Bonesetter App (Bonesetter Solutions LLC, Ann Arbor, Michigan, USA) was developed to address these gaps. Bernstein et al. previously published a paper using the Bonesetter App in axial alignment that records singular measurements. Coronal alignment requires multiple measurements and simple math to assess a lower extremity deformity, as opposed to the single measurements done in Bernstein et al.’s paper [6].

The purpose of this paper was to evaluate an online course teaching the skill “leg length and coronal deformity measurement” and to evaluate participants’ ability to demonstrate that skill on digital planning software for measuring deformity [7,8].

## Materials and methods

Participants

The participants included residents, fellows, and attendings from North America, South America, Europe, and Asia, all of whom were enrolled in the AO North America Trauma Osteotomy course. They registered for the course through email invitations. Although their specific levels of training were not identified, all participants were AO members. None of the participants had prior experience with the Bonesetter digital software.

Procedure

During the online AO North America Trauma Osteotomy course, participants were given standard instructions on how to use the digital planning software “Bonesetter” (Bonesetter App) and were given a lecture and tutorial on how to measure leg length discrepancy (LLD) and coronal alignment of the lower limb on standing long leg films. Instructions included LLD and the angular deformity in the femur, tibia, knee joint, and total deformity. Instructors were AONA faculty who were all well versed in online instruction and deformity surgery.

Measuring deformity using the Bonesetter App

Participants used the Bonesetter App to determine if the deformity was varus or valgus and if there was a deformity in the femur, tibia, or knee joint [9].

Measuring the deformity of the leg

To measure the length of the leg, participants used the line tool to draw a line from the hip center to the tibial plafond at the midpoint of the ankle. To assess the total deformity of the limb, an angle was drawn from the mechanical axis of the femur (from the center of the hip to the center of the knee) to the mechanical axis of the tibia (from the center of the knee to the center of the ankle). Participants determined whether the deformity was varus or valgus and identified its source: femur, tibia, or joint line.

Measuring the deformity of the femur

To measure the deformity of the femur, the participants used the angle tool to draw a line from the center of the femoral head to the center of the knee (the femoral mechanical axis) and a second line across the distal femoral joint line from the medial condyle to the lateral condyle, measured as the LDFA. The normal LDFA is 87 degrees, which indicates the deformity in the femur, which must be determined as varus or valgus (a positive number indicates valgus, and a negative number indicates varus).

Measuring the deformity of the joint

To measure the deformity of the joint (tibiofemoral), participants used the angle tool to draw a line across the distal femoral condyles and one across the proximal tibial joint line. The angle between the lines should be zero, but if there is an angle greater or less than zero, the joint is contributing to deformity and can be either varus or valgus.

Measuring the deformity of the proximal medial tibial angle (PMTAm)

To measure the PMTAm, an angle measurement was drawn from a line across the joint line of the tibia and a second line down the mechanical or anatomic axis of the tibia. The tibial deformity is calculated by subtracting the PMTAm from 87 (the normal PMTA). If the number is positive, this indicates varus, and if the number is negative, it indicates valgus.

Using this method, surgeons can determine how much of the total limb deformity is in the femur, tibia, or joint line. The digital planning software allows each participant to save their work so that the tutorial leader can evaluate it. In the online seminar, there were four cases with tutorials on how to measure coronal alignment. Participants’ measurements were then checked by two independent observers who were “super users.” Post-course analysis checked how many participants did the assignments, how many completed all components of the exercise, and how many did it correctly. A measurement was considered correct if the correct points were used to determine the measurement of an angle or length. Within the group of participants that completed all portions of the exercise correctly, a mean was established with the standard deviation to see how reproducible the measurements were with a large group of individuals. Errors and difficulties within the data were recorded and analyzed. An accurate sample exercise completed by the course leader was provided to the participants on video after a week, once they were allowed to complete the assignments. Participants could not change their saved materials once they were done, but several participants attempted the exercises multiple times. Only attempts completed before the answer key was released were used in the data analysis. Intraclass correlation coefficients were calculated using the ANOVA intraclass correlation on Microsoft Excel (Microsoft Corporation, Redmond, Washington, USA) and using the method described by analytics calculators [10].

## Results

Case 1 (deformity in the femur, tibia, and joint line)

A total of 176 participants completed the first exercise before the answer key was revealed. Among these, 121 (69%) answered all five questions correctly, while 55 (31%) provided incorrect answers. Measurements were deemed incorrect if the wrong points were used for angle measurements (Table 1).

**Table 1:** Data from those who completed the entire case and performed measurements correctly The standard deviation for the measurements was less than 1 degree for all participants who completed the exercise correctly, demonstrating the method’s reproducibility across participants.

Dataset	Standard deviation	Mean
Leg length	0.66 cm	76.39 cm
Total deformity	0.47 degrees	12.18 degrees
Joint	0.70 degrees	3.07 degrees
Femur	0.71 degrees	7.18 degrees
Tibia	0.72 degrees	2.3 degrees

For those participants who did not complete the exercise correctly, sources of error were found, including incorrect measurement of the total deformity (57.1%), incorrect measurement of the femur (5.71%), incorrect measurement of the joint (14.30%), and incorrect measurement of the tibia (5.71%). Additionally, some of the work was not understandable (2.86%), and some had all the measurements, but all measurement lines were deleted (14.28%).

**Figure 1:**
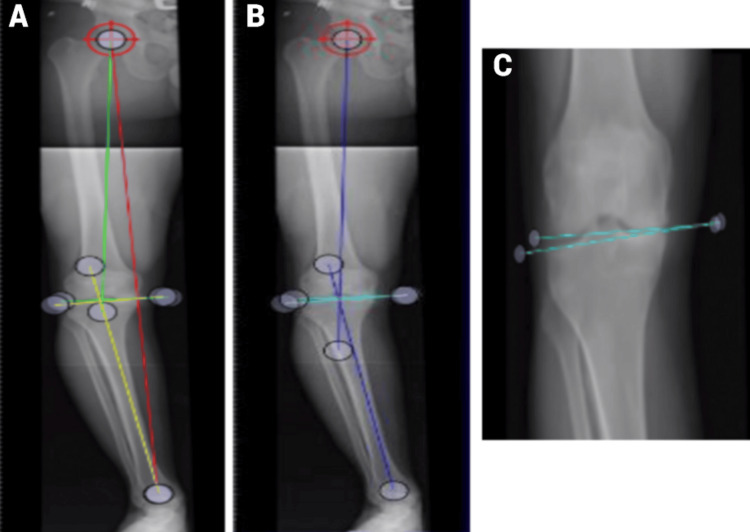
Standing images presented (Case 1) (A) The standing images presented in Case 1 include three different colored lines and a centering tool at the hip’s center. The red line denotes the leg length in centimeters, the green line represents the angle measurement for the femur, and the yellow line indicates the angle measurement for the tibia. This image does not show the total deformity or joint deformity angles. (B) This image, the same as the previous one, highlights only the total deformity and joint deformity. The dark blue line shows the total deformity angle, while the light blue/teal line represents the joint deformity angle. (C) This close-up view of the tibiofemoral joint shows the light blue/teal line marking the joint deformity angle, consistent with the previous image.

In Figure 1A-1C, the leg length was measured as 76.39 ± 0.66 cm. The total deformity was 12.18 ± 0.47 degrees varus, with 7.18 ± 0.71 degrees varus in the femur, 3.07 ± 0.70 degrees varus in the joint line, and 2.3 ± 0.72 degrees varus in the tibia.

Case 2 (varus femur deformity)

Out of the 145 data entries, 116 (80%) of all the participants completed all five data points correctly (Table 2).

**Table 2:** Data from those who completed the entire case and performed measurements correctly LLD, leg length discrepancy

Dataset	Standard deviation	Mean
LLD	1.49 cm	3.4 cm
Total deformity	0.61 degrees	15.54 degrees
Joint	0.73 degrees	2.11 degrees
Femur	1.25 degrees	12.29 degrees
Tibia	0.84 degrees	1.4 degrees

The sources of error included incorrect measurement of the total deformity (20%), incorrect measurement of the joint (58.1%), incorrect measurement of the tibia (12.9%), and incorrect left leg deformity measurement (3.23%). Additionally, some of the work was not understandable (3.23%), and some had all the measurements, but the measurement lines were deleted (3.23%).

**Figure 2:**
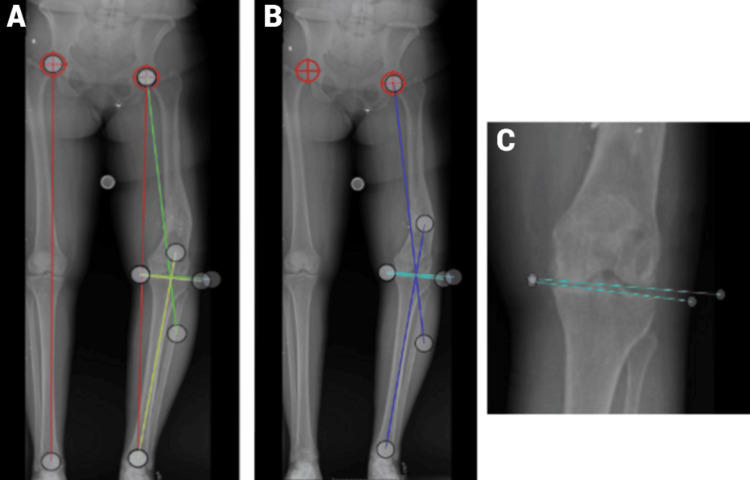
Standing images presented (Case 2) (A) This standing image displays both the right and left legs. The two targets on the acetabula indicate the hip centers. The red lines represent the length of the left and right legs in centimeters, which participants used to determine the length difference. The green lines denote the angle measurement for the femur, while the yellow lines indicate the tibial angle. (B) In this image, the focus is on the total deformity and joint deformity. The dark blue line shows the total deformity angle, and the light blue/teal line represents the joint deformity angle. (C) This close-up view of the joint illustrates where the angle measurement lines should be placed.

Most participants had a difficult time measuring the deformity in the joint (58.1%). Some participants did not measure the total deformity (19.4%), the deformity of the tibia, or the left leg deformity correctly. A few participants had work that was not understandable, with many unnecessary angle and length measurements (3.23%). There was a significant difference in correctness (p < 0.05) between Case 1 (57.1%) and Case 2 (80%). Compared to Case 1, more participants correctly measured the total deformity (Figure 2A-2C).

Case 3 (joint line deformity)

Out of the 52 data entries, 44 (83.8%) of all the participants completed all five data points correctly (Table 3).

**Table 3:** Data from those who completed the entire case and performed measurements correctly The standard deviation was less than 1 degree for all measurements except the joint measurement, where it was 1.44 degrees. LLD, leg length discrepancy

Dataset	Standard deviation	Mean
LLD	0.18 cm	0.24 cm
Total deformity	0.44 degrees	9.34 degrees
Joint	1.44 degrees	11.42 degrees
Femur	1.33 degrees	6.55 degrees
Tibia	0.77 degrees	-1.62 degrees

**Figure 3:**
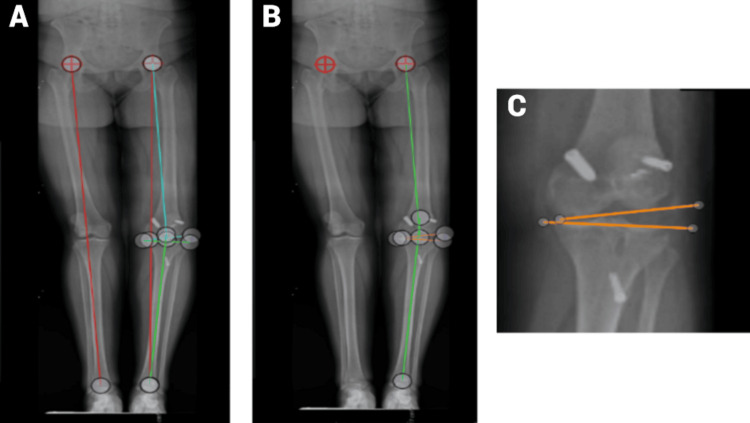
Standing images presented (Case 3) (A) In this standing image, the two red lines represent the length of the right and left legs in centimeters. The light blue/teal lines indicate the angle measurement for the femur, while the green lines show the angle measurement for the tibia. The two targets on both acetabula mark the hip centers. (B) In this standing scanogram, the green lines represent the angle measurements for the total deformity. The two orange lines indicate the angle measurements for the joint. (C) This closeup of the joint from the previous image illustrates the exact placement of the measurement lines.

In the 16.2% of participants who did not complete all five data points correctly, the sources of error included incorrect measurement of the joint (42.9%), incorrect measurement of total deformity (28.6%), and incorrect measurement of femoral deformity (28.6%). The images of where the lines should be exactly drawn are seen in Figure 3A-3C.

Case 4 (valgus femoral deformity)

Out of the 44 data entries, 34/42 (80.95%) of all the participants completed all five data points correctly (Table 4).

**Table 4:** Data from those who completed the entire case and performed measurements correctly The standard deviation of measurements was less than 1 degree for all parameters.

Dataset	Standard deviation	Mean
Right leg length	0.51 cm	95.68 cm
Right total deformity	0.79 degrees	15.62 degrees
Right joint deformity	0.63 degrees	0.61 degrees
Right femoral deformity	3.25 degrees	15.65 degrees
Right tibial deformity	0.86 degrees	1.93 degrees

**Figure 4:**
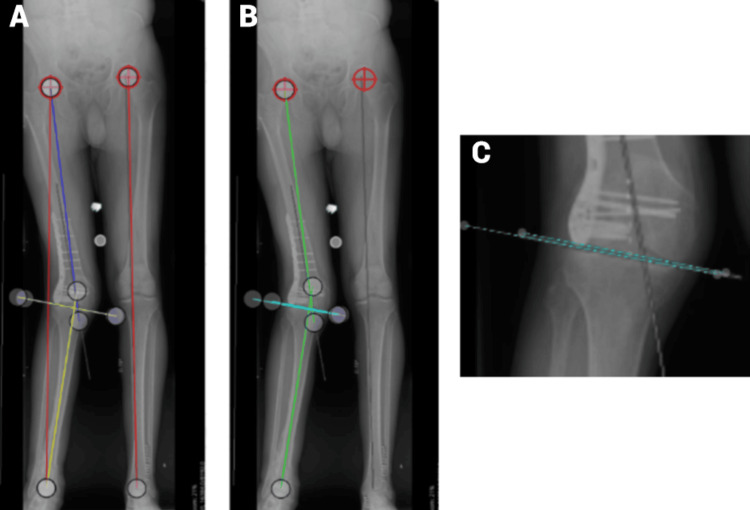
Standing images presented (Case 4) (A) This standing image shows the hip centers marked on both acetabula. The red lines indicate the length of the right and left legs in centimeters. The dark blue lines represent the angle measurements of the right femur, while the yellow lines denote the angle measurements of the right tibia. (B) This image displays the green lines for the angle measurements of the total deformity and the light blue/teal lines for the joint deformity. (C) This close-up focuses on the right joint, showing the minimal space between the femur and tibia.

In the 19% of participants who did not perform the exercise correctly, the sources of error included not measuring the leg length (50%) and incorrect measurement of the total deformity (50%). Figure 4A-4C shows the correct drawings of the angle measurements.

Overall

The number of participants that completed each case decreased from 176 to 145, 52, and 44 over the four cases. There was no method of penalizing or enticing the participants to do the exercises, as it was all based on the participant’s desire to learn.

## Discussion

The purpose of this study was to determine if an online orthopedic deformity course effectively taught how to measure lower extremity alignment. Overall, the session successfully demonstrated an increase in participants’ progress in measuring lower extremity deformity alignment when registered and monitored on the digital platform. There were no reported digital glitches during the exercise.

The rate of correctly completing the task for the four cases was 69% (Case 1), 81% (Case 2), 84% (Case 3), and 81% (Case 4). Out of the participants who failed the tasks, the most common errors were drawing the total deformity incorrectly, and often the rest followed. The incorrect measurement of the joint was also a common fault and may have been due to the very small measurement. In Case 1, 18% of total participants were unable to correctly calculate total deformity, suggesting there needs to be more time spent teaching this concept to participants and other orthopedic learners. Although instructors demonstrated the proper technique of leaving the lines in place to visualize the angles in the exercise, some participants deleted the lines, even with the correct answers. There were 18.1% of total participants who were unable to calculate the total deformity correctly (32/176 of people who did not complete the whole task correctly). Group 6, who had the answers correct but not the lines in place, was considered wrong because we had demonstrated to leave the lines in place to visualize the angles in the exercise. More emphasis should also be placed on this skill to ensure the proper visualization of angles and reduce the risk of potential errors in measurements. There were participants who consistently did not understand how to do the exercise. However, the advantage of this program is that people can be identified and retaught or have a secondary demonstration if they so wish.

The average standard deviation of length measurements across the exercises was +0.29 cm (244 measurements), and for angular measurements, it was +0.71 degrees (688 measurements) between participants. This was calculated from participants who correctly completed the tasks. It shows the accuracy of multiple individuals being able to get the same answer with this digital planning tool. The intraclass correlation coefficients were calculated for all four cases and were found to be (Case 1 = 0.999), (Case 2 = 0.982), (Case 3 = 0.961), and (Case 4 = 0.981), which translates to excellent reliability. The participants who participated throughout most cases improved their length lines and angle measurement lines.

The number of participants who attempted the four exercises was Case 1 = 100%, Case 2 = 82%, Case 3 = 30%, and Case 4 = 25%. The dropout rate from exercises one to four was considerable. This suggests that the online courses seem to have trouble keeping people engaged. Future courses can be made more interactive to increase the participatory rate or potentially offer physicians continuing medical education credits for completing the Bonesetter lecture.

The method of checking knowledge through exercises, such as Bonesetter, is a powerful educational tool because it confirms that unfamiliar tasks can be correctly performed when given proper instruction. As opposed to multiple choice questions, Bonesetter tests the specific skill that’s conveyed to the participants and assesses the accuracy of the skill performed. Being able to correctly perform the skill and correctly repeat it establishes the educational value of this module in the online program.

The study used the unique features of the Bonesetter App to evaluate orthopedic practitioners’ ability to measure coronal deformity after receiving online instruction. The clinical implications of the Bonesetter App are that more emphasis is placed on teaching, evaluating learning, discovering shortcomings immediately, and addressing or correcting them in the same course or venue. However, the problem with the online format is that there is little or no way to ensure participants complete the exercises as there is with face-to-face courses. There are still challenges to overcome when teaching skills online, but the usage of this Bonesetter App is evidence that online learning is heading in the right direction.

There are many online learning platforms that aim to assess the development process and knowledge of participants. The courses utilize tools such as the creation of online content, including videos, lab simulations, and quizzes, to assess the knowledge growth among the participants. One study developed and tested a six-week massive open online course on multiple sclerosis [11]. The participants who took this course found it to be beneficial as it improved their knowledge of multiple sclerosis and health literacy (knowledge tested through multiple-choice quizzes). Another study tested the effectiveness of a freely available hypertension online course on knowledge competencies for medical students in Uganda, and it was found that this self-paced online course improved knowledge on hypertension burden and management (knowledge tested through multiple choice quizzes) [12]. Additionally, an open online course was created and examined during the COVID-19 pandemic to determine if it was superior to conventional education in emergency nursing (knowledge tested through multiple-choice quizzes). It was found that comprehension of emergency nursing was stronger in the group of students participating in an online course that utilized an application than in the conventional education group [13]. Many of the studies that have implemented online platforms were created with the goal of assessing knowledge through multiple-choice quizzes. It is difficult to assess skills via an online platform. One course for dental students used an online hands-on course. The techniques used for this study promoted the learning of practical or surgical techniques on models such as bananas, pork bellies, or chicken thighs with live demonstrations (skills evaluated by an instructor via Zoom) [14,15]. Similarly, our study also attempted to assess the skill of measuring coronal deformity with a series of online lectures and video demonstrations. We assessed skill acquisition by evaluating exercises developed to test the skill. In our case, because there are definite correct measurements, we were able to assess if participants could reproduce the series of skills using the Bonesetter App.

Limitations

There are some limitations to this study. Post-analysis cannot determine how many participants already knew how to measure and calculate the deformity measurements required by the course. The exact level of medical training of each participant and whether they received or used other sources to understand deformity measurement are unknown. However, we do know that no participant had used the digital app Bonesetter App, as it had just been introduced. The selection bias in this study was limited by the fact that the participants were not chosen but instead were individuals who had heard about the course from a mass email that was focused on orthopedic interests, which included medical residents, fellows, and attendings. We did not separate them out, and as stated above, none of them had used the digital planning software. Information bias, meaning a lack of accurate measurements of key variables, is eliminated by the fact that all measurements are stored and accurately measured on the computer, so no individual can see or work with another individual due to the COVID-19 pandemic. After each case, the number of data entries decreased, which could also be a limitation. There were no confounding factors in the measurement aspect of this study, as the results are directly what were placed by the participants, and there is no way to change or modify them. Retention of the information by repeat testing later was not done and could be performed to see if the participants retained the information.

## Conclusions

The responses for total deformity, femoral deformity, tibial deformity, joint line deformity, and LLD were within one standard deviation of the examples provided by the instructor. The primary sources of error were related to measuring the total deformity and the deformity attributable to the knee joint. To address these issues, additional time should be allocated to ensure participants fully understand how to measure these parameters. The online tool was effective in identifying difficulties and allowing real-time corrections, benefiting both learners and educators.
